# Acupuncture for glucose and lipid metabolic disorders of polycystic ovarian syndrome: A systematic review protocol

**DOI:** 10.1371/journal.pone.0255732

**Published:** 2021-08-05

**Authors:** Yang Wu, Tao Peng, Yu Chen, Li Huang, Bisong He, Shaobin Wei

**Affiliations:** 1 Hospital of Chengdu University of Traditional Chinese Medicine, Chengdu, Sichuan, P.R. China; 2 Department of Traditional Chinese Medicine, Chengdu Second People’s Hospital, Chengdu, Sichuan, P.R. China; 3 Department of Gynecology, Hospital of Chengdu University of Traditional Chinese Medicine, Chengdu, Sichuan, P.R. China; Zhejiang University School of Medicine Women’s Hospital, CHINA

## Abstract

**Background:**

Polycystic ovary syndrome (PCOS) is a common gynecological disease that is often accompanied by some metabolic abnormality such as insulin resistance and dyslipidemia. As a non-pharmacological therapy, acupuncture is widely used for the treatment of PCOS, but the effectiveness for insulin resistance and lipid metabolic disorder remains controversial.

**Objectives:**

To assess the effectiveness and safety of acupuncture for insulin resistance and lipid metabolic disorder of women with PCOS.

**Search methods:**

Eight databases will be searched from inception to June 2021, three clinical trial registration platforms will be searched for relevant trials.

**Selection criteria:**

Randomized controlled trials (RCTs) of acupuncture therapy for insulin resistance and lipid metabolic of PCOS will be included.

**Data collection and analysis:**

Study screening, data collection, and analysis will be performed by two or more reviewers independently. We will calculate mean difference (MD), standard mean difference (SMD) with 95% confidence intervals (CIs). Data synthesis will be performed with RevMan V.5.3 software and with Stata V.15.0 software when necessary.

**PROSPERO registration number:**

CRD42020177846

## Introduction

### Description of the condition

Polycystic ovary syndrome (PCOS) is a common endocrine disorder in women of reproductive age which is characterized by anovulation, hyperandrogenism, polycystic ovarian morphology [1]. Nowadays, PCOS is considered to be a metabolic disease beyond the field of gynecology. Patients with PCOS have a higher rate of glucose and lipid metabolic disorders which can, in turn, aggravate the endocrine disorder. Studies have identified that women with PCOS have a high risk of diabetes with early-onset, metabolic syndrome and cardiovascular disease [2, 3].

Insulin resistance (IR) is the driving force behind glucose metabolic disorder, IR is considered to be a central link in the pathogenesis of PCOS which is highly associated with concomitant metabolic complications [4–6]. IR is defined as a condition of low insulin sensitivity which leads to compensatory hyperinsulinemia, it plays a major role in the pathogenesis of type 2 diabetes and the development of complications [7]. Hyperinsulinemia leads to an increase of androgen and luteinizing hormone secretion which in turn disturb glucose uptake and utilization and will lead to persistent anovulatory [8]. When dysfunctional pancreatic islet cells can not compensate for defective insulin secretion, it will develop to overt type 2 diabetes [7].

Women with PCOS are likely to accompany with IR and hyperinsulinemia, no matter obese or not [5]. Women with PCOS may exhibit lipid metabolic disorders even in the absence of obesity, although obese and overweight women with PCOS have a higher prevalence of IR compared to normal-weight women with PCOS [9]. Abnormal lipid metabolism in obesity, especially abdominal obesity, will further disrupt insulin signal pathways to aggravate IR. The study found that the lipid-stimulated cytokine responses following saturated fat ingestion may aggravate insulin resistance and increase androgen production [10]. Metabolic derangements induced by excess fat accumulation, and the elevation of free fatty acids and inflammatory adipocytokines play a crucial role in the development of insulin resistance [11, 12]. Thus, IR and abnormal lipid metabolism will result in a vicious circle of metabolic and endocrine disorders of PCOS.

For the treatment of PCOS patients with metabolic abnormality, metformin, a mild insulin sensitizer, is the first choice based on lifestyle adjustments and symptomatic treatments (eg, oral contraceptive, ovulation induction). Other common treatment options include pioglitazone and acarbose. However, there are also some limitations related to adverse effects and patient compliance with pharmacological treatments.

### Description of the intervention

Acupuncture is a popular form of Chinese medicine that has long been used in China. As a non-pharmacological therapy, acupuncture has gained increasing attention for the treatment of PCOS. Acupuncture has been demonstrated to improve menstrual frequency, decrease circulating testosterone and regulate sex hormones in PCOS with a favorable safety profile [13–16].In addition, acupuncture has beneficial effects on glucose and lipid metabolism and it was effective for weight loss [17–20]. Acupuncture is considered one of the treatment options for PCOS with glucose and lipid metabolic disorders.

### How the intervention might work

Multiple studies have found that acupuncture could improve symptoms of PCOS and regulate metabolic disorders through the coordination of multisystem, multitarget, and multidirectional effects [16, 21]. Acupuncture could modulate the function of the central nervous system and peripheral target organs [21]. Researches showed that acupuncture could regulate insulin secretion by regulating the insulin signaling pathway through the neuroendocrine pathway [22, 23]and regulate glucose and lipid metabolism of insulin target organs (eg, liver, adipose tissue, and skeletal muscle). Acupuncture can improve insulin resistance through modulation of adipocytokines to promote glucose and lipids metabolism and increase energy consumption [24].

### Why it is important to perform this review

PCOS is a chronic disease that requires continuous and prolonged treatment, which is a challenge for both patients and drugs. Gastrointestinal side-effects associated with metformin intolerance may occur in some PCOS patients including nausea, gastric discomfort, and diarrhea [25]. Long-term use or overdose of metformin may cause liver injury or kidney problems [26]. Some patients are nonadherent to long-term oral medication and are seeking for nonpharmacological treatments.

Acupuncture as one of the main therapies for complementary and alternative medicine has received increasing attention. But there is still a controversy about the effectiveness of acupuncture for PCOS women with IR and lipid metabolic disorder. A small number of systematic reviews have been conducted to assess the effectiveness of acupuncture for IR. But different types of diseases were included [27], and data from different types of interventions were merged [28], which may lead to a controversial conclusion. Another study only compared acupuncture alone versus placebo or standard therapy [29], ignored combination therapies of acupuncture and others. However, combination therapies are commonly used in clinical practice and clinical trials. Add these types of studies, we can explore that whether the combination has a synergetic effect with each other. Besides recent researches have been constantly performed to observe the effect of acupuncture in treating glucose and lipid metabolism of PCOS [30, 31]. Therefore, we aim to conduct a systematic review of acupuncture for glucose and lipid metabolic disorder of PCOS and hope to provide an objective basis for healthcare practitioners and health policymakers that will benefit patients.

### Objectives

This systematic review aims to evaluate the safety and effectiveness of acupuncture for IR and lipid metabolic disorders of PCOS patients.

## Methods

### Study registration and design

The systematic review protocol has been registered prospectively in the International Prospective Register of Systematic Reviews (PROSPERO) (CRD42020177846). This protocol is reported in line with the Preferred Reporting Items for Systematic Review and Meta-Analysis Protocols (PRISMA-P) statement guidelines [32]. The PRISMA-P checklist is shown in the S1 Appendix.

And the study will be performed according to the Cochrane Handbook for Systematic Reviews of Interventions and presented based on Preferred Reporting Items for Systematic Reviews and Meta-analyses (PRISMA) guidelines [33].

### Criteria for including studies in this review

#### Types of studies

Randomized controlled trials (RCTs) of acupuncture therapy for IR and lipid metabolic of PCOS will be included. Summary results of ongoing and completed trials published on the clinical trial registration platform will also be included. The sample size of both experimental and control groups for each individual included trial should be larger than 20 respectively.

#### Types of participants

Participants who were diagnosed with PCOS according to the ESHRE and ASRM consensus in Rotterdam in 2003, will be included regardless of their age, race, and background. The Rotterdam consensus required at least 2 out of 3 criteria including 1) Oligo-and/or anovulation, 2) Clinical and/or biochemical signs of hyperandrogenism, 3) Polycystic ovaries, and exclusion of other aetiologies (such as congenital adrenal hyperplasias, androgen-secreting tumors, Cushing’s syndrome) [1].

If the trials did not use the Rotterdam consensus, but the diagnostic criteria were clearly stated, we will evaluate the diagnostic criteria to confirm whether they meet the Rotterdam consensus. The authors will be contacted to obtain clarification if there is insufficient information. If clarification is not available, the trials will be excluded.

#### Types of interventions

*Experimental interventions*. The interventions considered in the review were described as acupuncture, which is defined as needles be inserted into acupoints, pain points, or trigger points. The interventions including manual acupuncture, electro-acupuncture, combined acupuncture with moxibustion, acupuncture combined with lifestyle management (such as exercise, control diet), or acupuncture combined with metformin. Trials in which any one of the above treatments combined with a basic treatment (such as Diane-35, clomiphene) which is used in control groups, will also be included.

Other stimulations to specific points such as catgut embedding, point injections, or acupoints stimulation without needle insertion (such as acupressure, massage) will be excluded. Trials in which patients have previously been treated with insulin sensitizer or insulin secretagogues before the study started will be excluded.

*Control interventions*. Eligible comparators were placebo, sham acupuncture, no treatment, lifestyle management, metformin, and metformin combined with sham acupuncture or lifestyle management. And any one of the above treatments combined with the same basic treatment(such as Diane-35, clomiphene), will also be included.

Studies only compared different forms or methods of acupuncture, and compared acupuncture with a different type of Chinese Medicine (such as Chinese herbs) will be excluded.

#### Types of outcome measures

*Primary outcome*. (1)Homeostasis model assessment of insulin resistance (HOMA-IR) [34]. HOMA-IR = FINS(μU/ml) × FPG(mmol/L)/22.5. Homeostasis model assessment β cell function (HOMA-β). HOMA-β = 20× FINS(μU/ml) / (FPG(mmol/L)−3.5).

(2)Serum triglyceride (TG).

*Secondary outcomes*. (1)Glucose metabolic measures: The ratio in the area under the curve (AUC) of insulin and glucose during the oral glucose tolerance test (OGTT): AUC_INS_/AUC_GLU_. Area under the curve of glucose, insulin and C-peptide: AUC_GLU_, AUC_INS_, AUC_C-P_.

(2)Lipid metabolic measures: Serum total cholesterol(TC), Serum high-density lipoprotein cholesterol(HDL-C), Serum low-density lipoprotein cholesterol(LDL-C).

(3)Anthropometric measures: Body mass index(BMI), Waist circumference(WC), Waist-to-hip ratio(WHR).

(4)Adverse events related to acupuncture.

### Search methods for identification of studies

#### Electronic searches

The following databases will be searched from inception to June 2021, regardless of the publication status: Cochrane library, PubMed, EMBASE, Web of Science, China National Knowledge Infrastructure (CNKI), Chinese Biomedical Literature Database (CBM), Wan-Fang database, Chinese Scientific Journal Database (VIP). There is no language restriction.

The search terms consisted of four parts: population (PCOS), intervention (acupuncture), outcome (eg, HOMA, glucose metabolism, lipid metabolism, glycolipid metabolism), study design (RCT). Searches will combine medical subject headings terms and free words in title and abstract. The search strategy for PubMed is shown in the S2 Appendix.

#### Searching other resources

RCTs will also be obtained from the reference lists of relevant studies and published systematic reviews. The ClinicalTrials.gov, the Chinese clinical Trial Registry, and the WHO International Clinical Trial Registry Platform will be searched for ongoing or unpublished trials.

### Data collection and analysis

#### Selection of studies

The search results will be imported into Endnote X9. After removing duplicate records, titles and abstracts will be checked to identify applicable studies by two independent reviewers (BH and TP). Then the full texts will be read for further selected according to the inclusion criteria. Excluded studies will be listed with reasons. Any disagreements will be resolved by discussion. The flow diagram of the study selection process is shown in the S1 Fig.

#### Data extraction and management

Information will be extracted from the included studies by two independent reviewers (BH and TP) using a data extraction form in excel, which included citation information (title, authors, source of publication, publication year, country, sponsor), study methods (design, sample size, method of randomization, allocation concealment, blinding), participant characteristics (age, diagnostic criteria), intervention details (type of acupuncture/control, treatment duration, treatment frequency), results (outcome measures, adverse events), and so on. Any discrepancy in the process of cross-checking will be resolved by consensus.

Original authors will be contacted twice by email using an institutional email account over 4 weeks if essential data are insufficient or not reported in a usable format. When some raw data were not available from authors, it can be acquired based on available data referring to advanced methods of data extraction [35].

#### Risk-of-Bias assessments

Two independent reviewers (YC, YW) will evaluate the risk of bias using the following domains described in Cochrane’s tool: random sequence generation, allocation concealment, blinding of participants and personnel, blinding of outcome assessment, incomplete outcome data, selective reporting, and other bias. The risk of bias for each domain of RCTs will be classified as low risk, high risk, and unclear risk. Any disagreement will be resolved by consultation among the reviewers (LH, SW).

Meanwhile, the methodological quality of each included trial will be classified as high quality, low quality, or moderate quality base on the following criteria according to Zhao et al. [36]: (1) trial will be considered high quality when both randomization and allocation concealment are assessed as low risk of bias, and all other items are assessed as low or unclear risk of bias; (2) trial will be considered low quality if either randomization or allocation concealment is assessed as high risk of bias, regardless of the risk of other items; (3) trial will be considered moderate quality if they do not meet criteria for high or low risk.

#### Measures of treatment effect

RevMan V.5.3 will be used for data synthesis and analysis. For the outcomes are continuous data, the mean difference (MD) will be used to assess the treatment effect with 95% CIs if the studies used the same measurement scales. And the standard mean difference (SMD) will be used if different scales were used.

It is possible that individual studies may consist of multiple groups [37], such as different non—acupuncture control interventions (eg, placebo, sham acupuncture, or no treatment). We will combine the groups from multiple arm studies into a single group according to the data merging methodology of subgroups before data synthesis.

#### Dealing with missing data

Original authors of the trial will be contacted for the relevant missing data. If missing data cannot be obtained, an imputation method will be used [32, 35]. We will conduct sensitivity analysis to assess the impact on the overall treatment effects. And the potential impact of the effect of missing data on the overall treatment effects of the review will be addressed in the discussion.

#### Assessment of heterogeneity

Statistical heterogeneity between different trials will be evaluated by the I^2^ statistic with Q statistic test and visual inspection of forest plots. The I^2^ statistic of 25%, 50%, and 75% indicates low, medium, and high heterogeneity, respectively [38]. I^2^ value > 50% indicates significant heterogeneity. When I^2^ value > 50%, clinical, methodological, or statistical heterogeneity will be assessed to find possible sources of heterogeneity firstly. If there is clinical or methodological heterogeneity, subgroup analysis or meta-regression analyses (with Stata V.15.0 software) will be used to explore the source of heterogeneity. If there is statistical heterogeneity, data will be pooled based on random-effects model. And sensitivity analysis will also be performed to investigate the influence of every single study on the overall analyses. If there is significant heterogeneity among trials and cannot be explained, a descriptive analysis will be performed instead of a meta-analysis.

#### Assessment of reporting biases

A funnel plot, as well as statistical tests (Egger test and Begg test by Stata V.15.0 software), will be used to assess reporting bias if more than 10 trials are included for meta-analysis [39, 40].

#### Data synthesis

Data synthesis will be performed with the RevMan software (V.5.3) according to Cochrane Handbook for Systematic Reviews of Interventions if studies are sufficiently homogeneous. The Inverse Variance (IV) method and random-effects model with 95% CI will be used to calculate a pooled estimate of treatment effect since all the prespecified outcomes are continuous variables and a certain degree of heterogeneity is expected among trials that will be included.

However, if quantitative synthesis is not appropriate, a systematic narrative synthesis will be provided to summarize and explain the characteristics and findings of the included studies [32].

#### Subgroup analysis

We plan to conduct subgroup analysis based on different types of experimental intervention, control group, and different methodological quality. If there is still significant heterogeneity, the source of heterogeneity will be explored and trials will be further classified to perform subgroup analysis.

#### Sensitivity analysis

Sensitivity analysis will be performed to identify whether the overall results and conclusions are affected by different criteria: characteristics of participants, sample size, methodological quality, statistical model (RE or FE model). Leave-one-out sensitivity analysis will be performed to check the influence of a single study. Sensitivity analysis will also be performed to explore the source of heterogeneity.

#### Summary of evidence

The quality of evidence for all outcomes will be judged using the Grading of Recommendations Assessment, Development and Evaluation working group methodology [32]. It will be adjudicated as ‘high’, ‘moderate’, ‘low’ or ‘very low’ based on: risk of bias, inconsistency, indirectness, imprecision, publication bias, and additional domains [41]. The results will be presented in ‘summary of findings’ tables.

### Patient and public involvement statement

No patient involved.

### Ethics and dissemination

This review will not involve private information from individuals and will not affect patient rights therefore does not require ethical approval. The results of this review will be disseminated through peer-reviewed publications and conference reports.

## Discussion

This meta-analysis is expected to provide objective evidence of acupuncture for insulin resistance and lipid metabolic disorder of PCOS and provide a convincing conclusion of whether acupuncture combined with metformin is more effective than metformin alone. This evidence may help patients, healthcare practitioners, and health policymakers who are seeking for complementary and alternative therapy.

## Supporting information

S1 Appendix The PRISMA-P checklist. (DOCX)

S2 Appendix The search strategy for PubMed. (DOCX)

S1 Fig The PRISMA flow diagram of the study selection process. (TIF)
