# Supplementary material for: Acupuncture for glucose and lipid metabolic disorders of polycystic ovarian syndrome: A systematic review protocol
Source: PLoS One. 2021 Aug 5;16(8):e0255732. doi: 10.1371/journal.pone.0255732 (PMC8341540; doi:10.1371/journal.pone.0255732)
Supplement: S2 Appendix — (DOCX) [file pone.0255732.s002.docx]

**S2 Appendix: The search strategy for PubMed**

| #1 | "Polycystic Ovary Syndrome"[MeSH Terms] |
| --- | --- |
| #2 | "PCOS"[Title/Abstract] OR "stein leventhal syndrome"[Title/Abstract] OR "sclerocystic ovar*"[Title/Abstract] OR "polycystic ovar*"[Title/Abstract] |
| #3 | #1 or #2 |
| #4 | "Acupuncture Therapy"[MeSH Terms] |
| #5 | "acupuncture"[Title/Abstract] OR "needling"[Title/Abstract] OR "electroacupuncture"[Title/Abstract] OR "electro-acupuncture"[Title/Abstract] |
| #6 | #4 or #5 |
| #7 | "insulin resistance"[MeSH Terms] OR "insulin sensitivity"[Title/Abstract] OR "HOMA"[Title/Abstract] OR "OGTT"[Title/Abstract] |
| #8 | ("Glucose"[Title/Abstract] AND "lipid metaboli*"[Title/Abstract]) OR "glycolipid metaboli*"[Title/Abstract] |
| #9 | "glucose metaboli*"[Title/Abstract] OR "sugar metaboli*"[Title/Abstract] OR "Glycometabolism"[Title/Abstract] OR "glycogen disease"[Title/Abstract] OR "Saccharometabolism"[Title/Abstract] |
| #10 | "lipid metabolism"[MeSH Terms] OR "lipid metaboli*"[Title/Abstract] OR "fat metaboli*"[Title/Abstract] |
| #11 | #7 OR #8 OR #9 OR #10 |
| #12 | "randomized controlled trial"[Publication Type] OR "controlled clinical trial"[Publication Type] OR "randomized"[Title/Abstract] OR "randomized"[Title/Abstract] OR "placebo"[Title/Abstract] OR "drug therapy"[MeSH Subheading] OR "randomly"[Title/Abstract] OR "trial"[Title/Abstract] OR "groups"[Title/Abstract] |
| #13 | "animals"[MeSH Terms] NOT "humans"[MeSH Terms] |
| #14 | #12 NOT #13 |
| #15 | #3 OR #6 OR #11 OR #14 |
