# Supplementary figures and images for: Acupuncture for glucose and lipid metabolic disorders of polycystic ovarian syndrome: A systematic review protocol
Source: PLoS One. 2021 Aug 5;16(8):e0255732. doi: 10.1371/journal.pone.0255732 (PMC8341540; doi:10.1371/journal.pone.0255732)

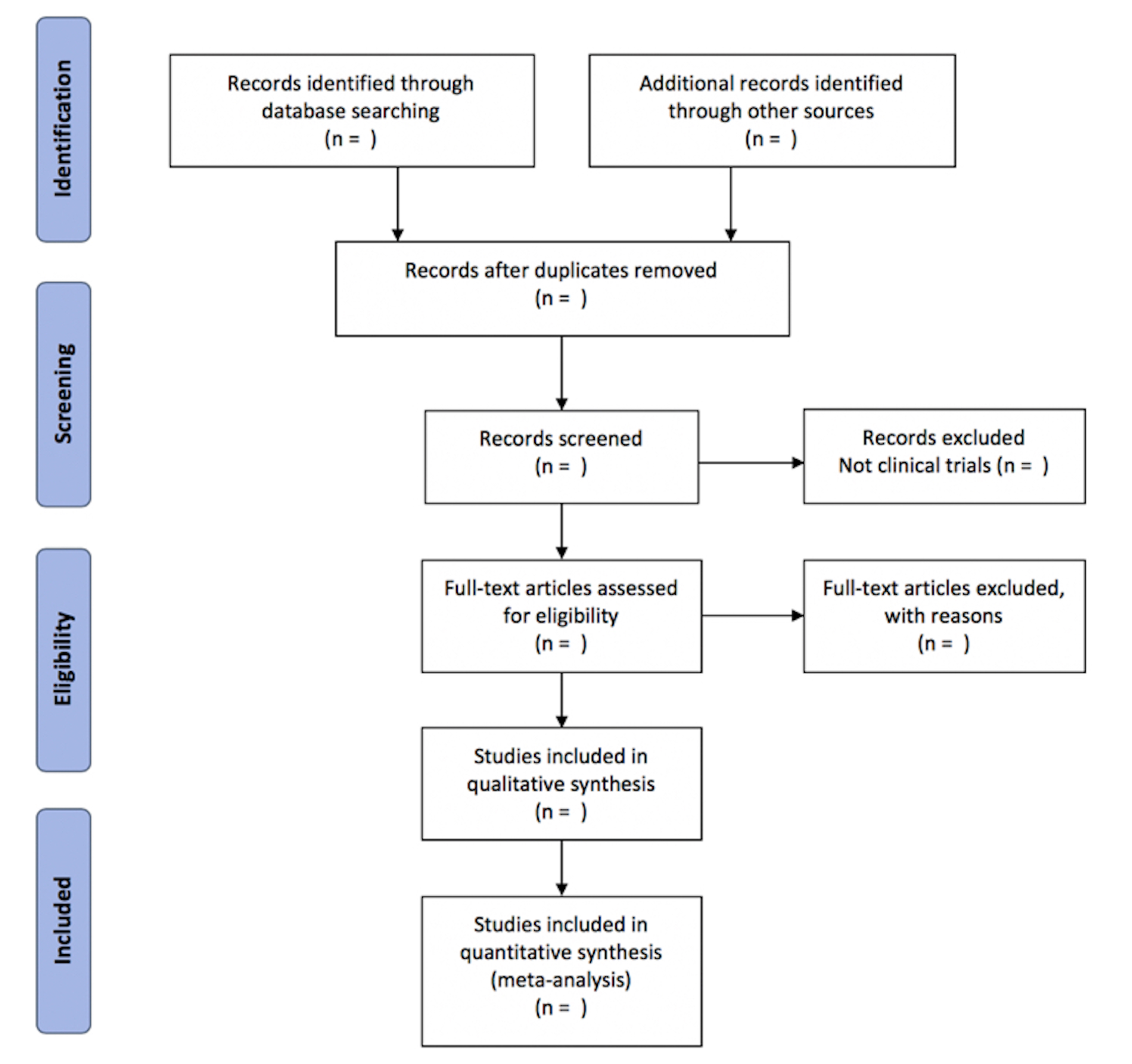

Supplement: S1 Fig — (TIF) [file pone.0255732.s003.tif]
